# The Association of the One-Abutment at One-Time Concept with Marginal Bone Loss around the SLA and Platform Switch and Conical Abutment Implants

**DOI:** 10.3390/jcm11010074

**Published:** 2021-12-24

**Authors:** Nasreen Hamudi, Eitan Barnea, Evgeny Weinberg, Amir Laviv, Eitan Mijiritsky, Shlomo Matalon, Liat Chaushu, Roni Kolerman

**Affiliations:** 1 Private Practice, Calanswa 40640, Israel; hnasreen10@gmail.com; 2 Private Practice, Tel Aviv 6416304, Israel; eitanbarnea@gmail.com; 3 Department of Periodontology and Oral Implantology, The Maurice and Gabriela Goldschleger School of Dental Medicine, Tel Aviv University, Tel Aviv 6997801, Israel; evgenywein@gmail.com (E.W.); Liat.natanel@gmail.com (L.C.); 4 Department of Oral Biology, The Goldschleger School of Dental Medicine, Tel Aviv University, Tel Aviv 6997801, Israel; 5 Department of Maxillofacial Surgery, The Maurice and Gabriela Goldschleger School of Dental Medicine, Sackler School of Medicine, Tel Aviv University, Tel Aviv 6997801, Israel; laviv@tauex.tau.ac.il; 6 Department of Otolaryngology, Head and Neck Surgery and Maxillofacial Surgery, Tel Aviv Sourasky Medical Center, Sackler School of Medicine, Tel Aviv University, Tel Aviv 6139001, Israel; mijiritsky@bezeqint.net; 7 Department of Oral Rehabilitation, The Maurice and Gabriela Goldschleger School of Dental Medicine, Tel Aviv University, Tel Aviv 6997801, Israel; matalon@tauex.tau.ac.il

**Keywords:** connection and disconnection abutment, implant bone loss, implant neck, marginal bone level, platform switch

## Abstract

Objectives: Repeated abutment disconnection/reconnection may compromise the mucosal barrier and result in crestal bone level changes. The clinical significance of this phenomenon is not yet clear, as most studies on this topic are short-term. Therefore, the aim of the present study was to evaluate the influence of abutment disconnections and reconnections on peri-implant marginal bone loss over a medium-term follow-up period. Material and methods: Twenty-one patients (6 men and 15 women) with a mean age 66.23 ± 9.35 year at the time of implant placement were included. All patients who received two adjacent nonsubmerged implants were randomly assigned into one of the two groups: definitive multiunit abutments (DEFs) connected to the implant that were not removed (test group) or healing abutments (HEAs) placed at surgery, which were disconnected and reconnected 3–5 times during the prosthetic phase (control group). Peri-implant marginal bone levels (MBL) were measured through periapical X-rays images acquired immediately after the surgery (baseline), at 4–7 months immediately after prosthetic delivery, and at 1-year and 3-year follow-up visits. Results: No implant was lost or presented bone loss of more than 1.9 mm during the 3-year follow-up; thus, the survival and success rate was 100%. Peri-implant mucositis was noticed in 38.1% DEFs and 41.9% of HEAs at the 3-year follow-up assessment. At the end of 3 years, the MBL was −0.35 ± 0.69 mm for participants in the DEFs group and −0.57 ± 0.80 mm for the HEAs group, with significant statistical difference between groups. Conclusions: Immediate connection of the multiunit abutments reduced bone loss in comparison with 3–5 disconnections noted in the healing abutments 3 years after prosthetic delivery. However, the difference between the groups was minimal; thus, the clinical relevance of those results is doubtful.

## 1. Introduction

The peri-implant mucosa around titanium implants has been studied in a series of preclinical and clinical studies [1,2,3,4,5,6,7,8,9,10,11,12]. It was observed that the implant–mucosal barrier comprises two components: junctional epithelium (JE) of 2 mm long and connective tissue compartment of 1–1.5 mm height [2]. It was suggested [2] that this attachment serves the purpose of protecting the zone of osseointegration from factors released from the plaque and the oral cavity. Since the junctional epithelium consistently terminated at a certain level during healing, it was suggested that there was an interaction between the junctional epithelium and the titanium dioxide of the abutment surface and that this zone of “interaction” was not recognized as a wound. However, any disturbance of the zone of “connective tissue integration” may affect marginal peri-implant tissues including peri-implant bone [3]. The choice of specific material and surface treatment of the transmucosal portion used for rehabilitation can impact the long-term outcomes of dental implants [4]. The main advantage of transmucosal portion with a smooth machined surface is its capacity to reduce the risk of bacterial colonization and inflammation, thus, reducing the incidence of peri-implantitis [4]. Concomitantly, several in vivo studies concluded that rough/modified surfaces healing abutments may promote the presence of adhesion molecules such as integrins [5] and the expression of collagen-associated genes compared to machined surfaces [6]. Moreover, connective tissue fibers were found to be denser and perpendicularly oriented to the surface around microgrooved abutments, whereas in the machined titanium abutments, the fibers were oriented parallel to the abutment surface [4]. The transmucosal profile (flat or concave) may also influence bone resorption and improve connective tissue attachment around implants [7].

Bone remodeling around implants may be affected by other factors such as microgaps causing microleakage between the implant and abutment [8], micromovement at the implant–abutment interface [9], and abutment disconnection and/or reconnection [10,11,12]. However, the establishment of the peri-implant mucosal complex did not seem to be influenced by the implant type or mode of healing when comparing nonsubmerged versus submerged dental implants [13,14].

Previous animal studies have pointed to a reduced epithelial component at two-piece implants with platform-switching [15,16]. A circumferential mismatch of 0.3–0.5 mm, generated by the connection of diameter-reduced titanium abutments (TA) relative to the implant shoulder (IS), was able to decrease the vertical dimension of JE over an observation period of up to 6 months [15,16]. Moreover, in vivo studies using platform-switching designed restorations showed that crestal bone levels may be preserved as previously confirmed by the results of a systematic review [17]. On the other hand, repeated abutment disconnection/reconnection may compromise the mucosal barrier and result in crestal bone level changes as found in a preclinical study [18]. Later, Canullo et al. used the term “one abutment at one time” (AOT) concept to refer to the connection of an immediate nonremoval abutment [12]. Although some studies confirmed the advantages of the AOT concept [10,11,12,19], others found no differences in bone loss when immediate positioning of a definitive abutment was compared to that of a repeated abutment disconnection [20].

The purpose of this prospective clinical study was to compare crestal bone resorption around implants restored using the one-abutment one-time protocol with multiunit abutment versus implants connected to healing abutment that were reconnected 3–5 times.

We hypothesized that high survival and success rates are achieved, and no difference found between multiunit to healing abutment regarding marginal bone level.

## 2. Materials and Methods

The study was approved by the Ethics Committee of Tel Aviv University, No. 0002277-1. The study consisted of all consecutive patients, treated by two experienced specialists (more than 10 years) (E.B and R.K.) during the years 2015 to 2017. All patients signed an informed consent form and allowed the use of their clinical data.

Inclusion criteria:

1. Age of at least 21 years.
2. Good oral hygiene was defined as a full mouth plaque score <25% [21].
3. Partial edentulism with the need for two adjacent implant-supported fixed restorations.
4. Treated and stable periodontal disease of the remaining dentition.
5. Availability of native healed bone to accommodate at least two adjacent implants of ≥8 mm length and ≥3.75 mm width without bone augmentation and a minimum of buccal/lingual or palatal wall of 2 mm after implant installation.
6. Existence of a sufficient amount of (≥2 mm) keratinized gingiva for transgingival healing.
7. The presence of opposing dentition (natural or restored).

Exclusion criteria:

1. Uncontrolled diabetes, untreated malignancies, pregnancy, previous/current bisphosphonate therapy, immune diseases.
2. Previous radiation therapy to the head and neck area.
3. Untreated pathology in the jaws.
4. Psychological problems.
5. Oral mucosal diseases, such as lichen planus.
6. Poor oral hygiene (defined as full mouth plaque score >25% at re-evaluation) or lack of compliance with treatment visits or protocol.
7. Active periodontal disease involving the residual dentition.
8. Need for bone augmentation.
9. Light/heavy smokers.

## 3. Treatment Protocol

*Preliminary treatment*. A schematic outline of the study is presented in Table 1. All patients underwent a comprehensive thorough presurgical evaluation including demographic data, smoking habits, periodontal chart (and periodontal diagnosis), occlusal analysis, full mouth periapical radiographs, and CT scans prior to implant placement. The patients underwent cause-related therapy including oral hygiene instructions and training and scaling and root planning (SRP) wherever indicated. If necessary, in re-evaluation, patients had additional periodontal therapy (periodontal surgery or repeated SRP) aimed at reducing periodontal probing depth and bleeding on probing and further improving plaque control to achieve a hygiene index (HI) below 25% [21].

### 3.1. Surgical Technique and Postoperative Management

One hour prior to surgery, premedication was administered, including 8 mg dexamethasone (Aspen, Dublin, Ireland) and antibiotics—875 mg amoxycillin–clavulonate potassium (Augmentin, Smith Kline, Brentford, UK) or 600 mg clindamycin HCl (Dalacin-C, Pfizer NV/SA, Puurs, Belgium) for penicillin-allergic patients. Immediately before the procedure, patients performed a one-minute rinse with 0.2% chlorhexidine solution (Tarodent mouthwash, Taro Pharmaceutical Industries, Haifa, Israel).

**Figure 1:**
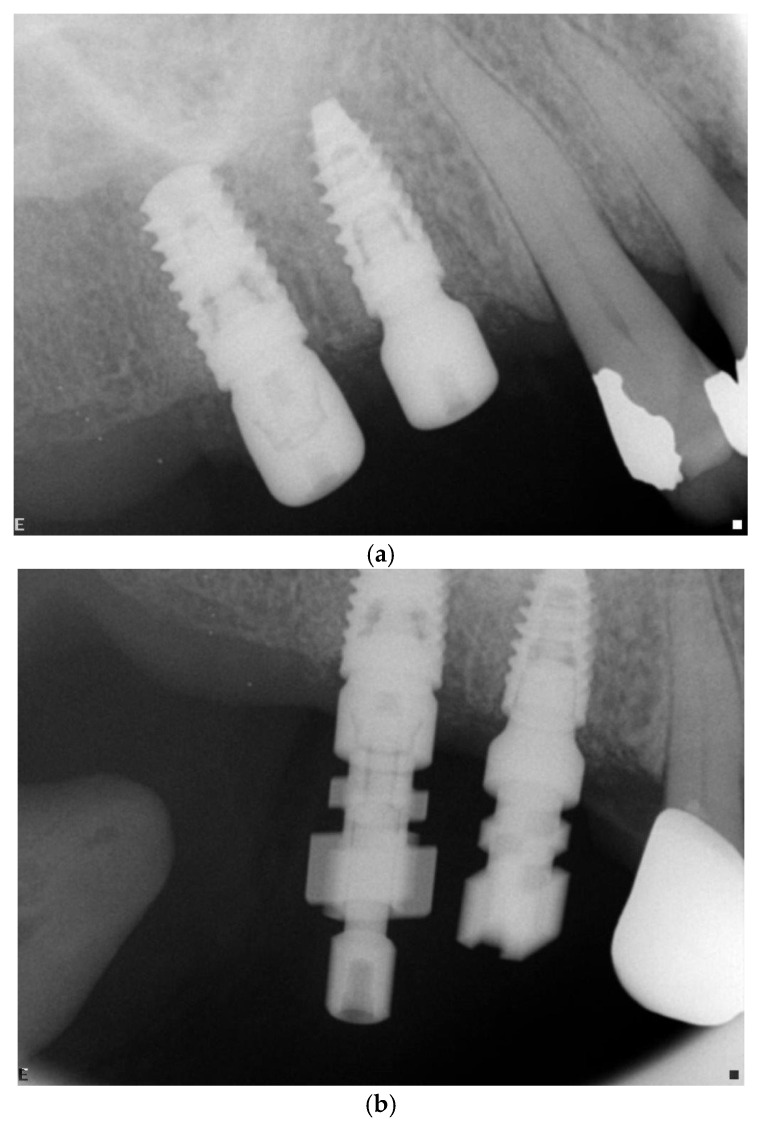

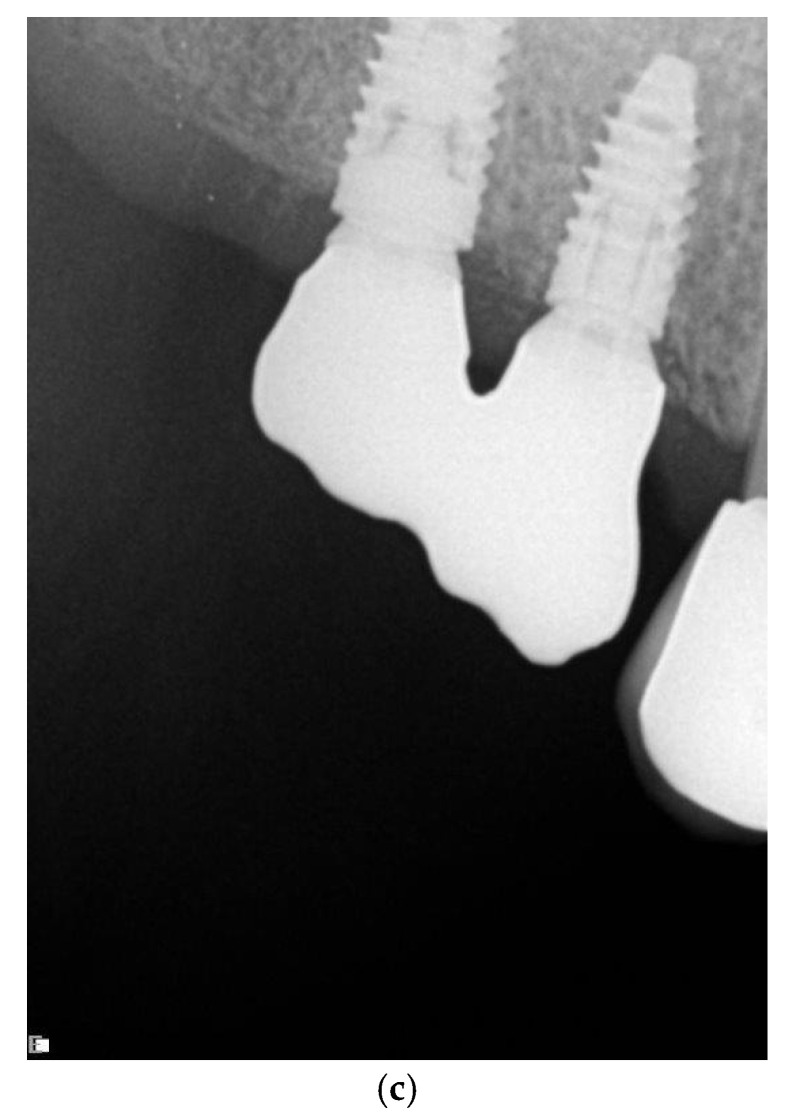
(**a**–**c**) Maxilla: (**a**) right upper first premolar and first molar replaced by implants; (**b**) impression copings of multiunit and healing abutments at 24 weeks; (**c**) 3-year follow-up.

**Figure 2:**
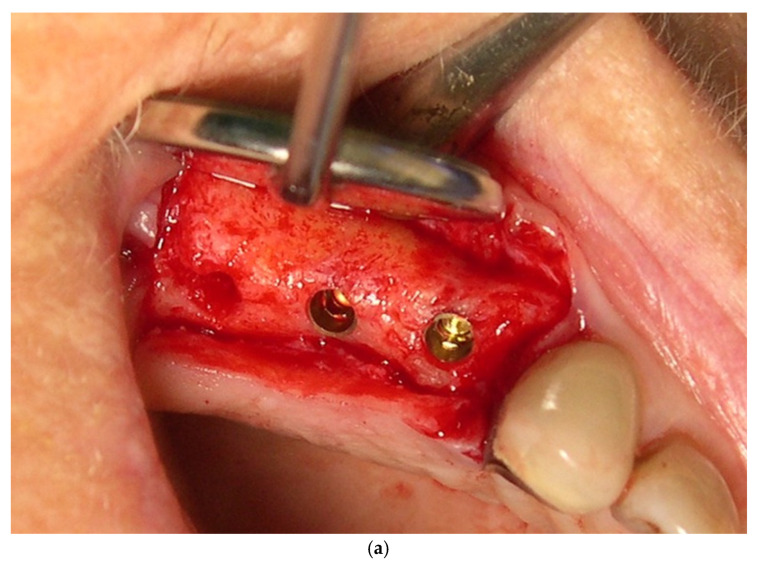

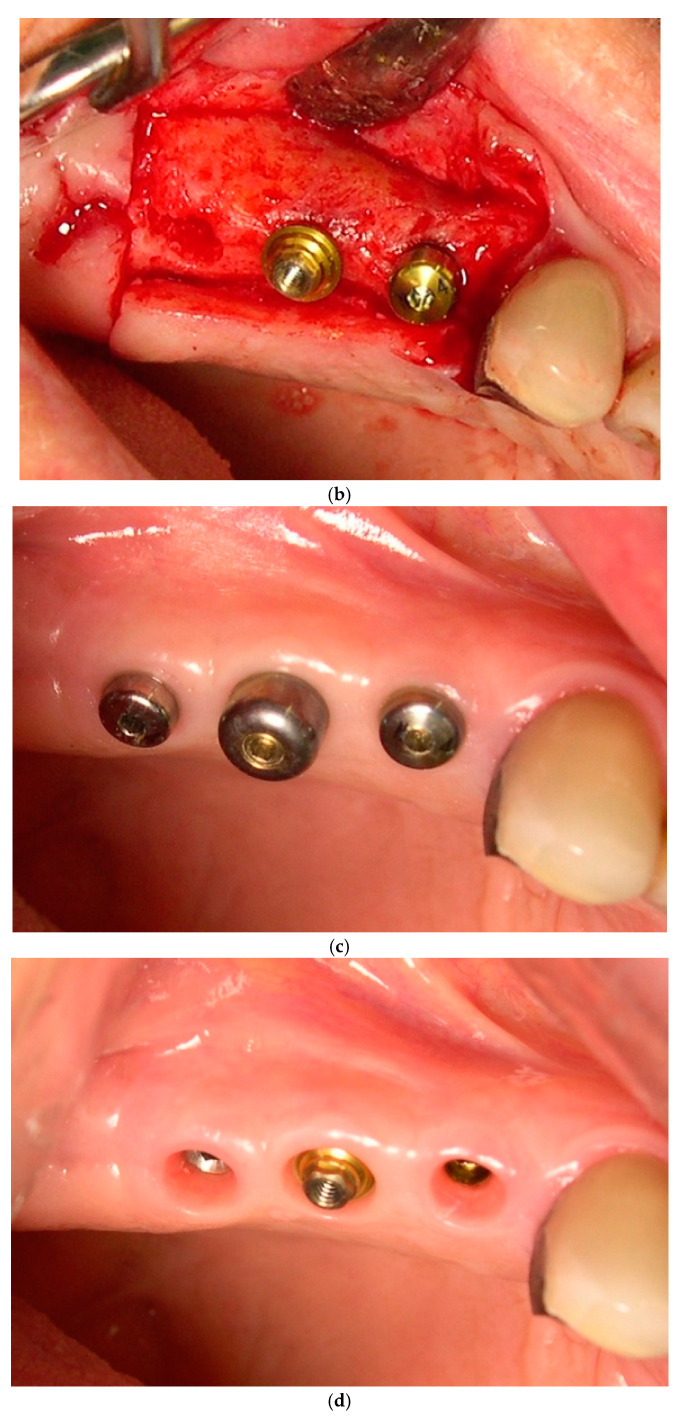

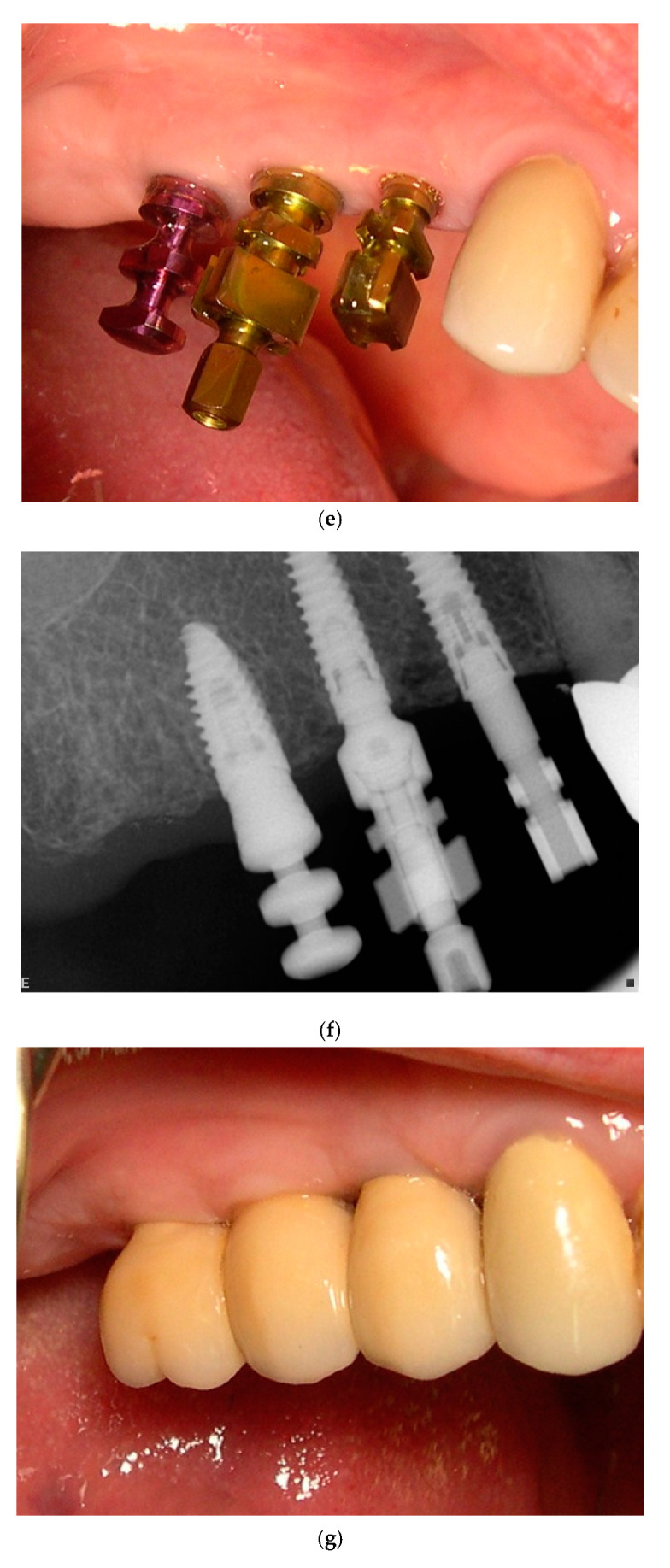

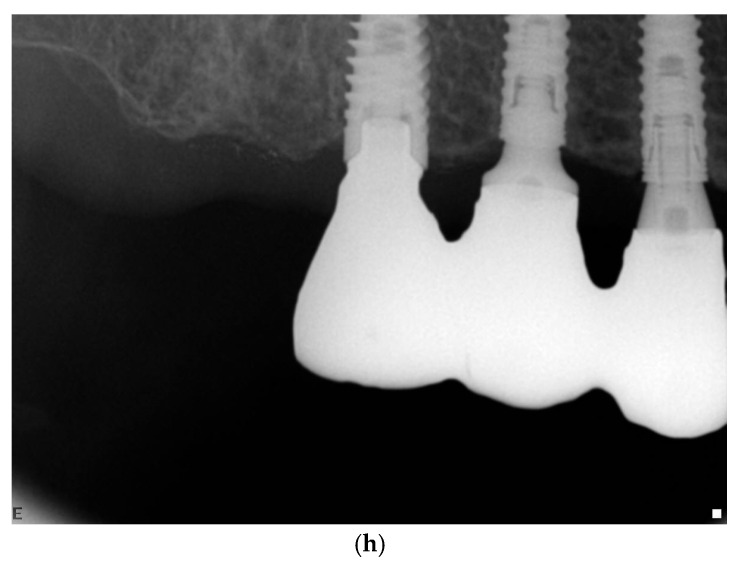
(**a**–**h**) Maxilla: (**a**) Two implants replacing missing right first and second premolars in an 83-year female. The implants shoulder was flushed with the bone. (**b**) Multiunit (test) and healing (control) abutments were connected after final seating of the implants. (**c**) Twenty-four weeks after placement, healthy peri-implant keratinized gingiva. (**d**) Healthy sulcular epithelium at both test (second premolar) and control (first premolar). (**e**) Transfer adaptation for “open tray “impression technique. (**f**) Radiographic transfer adaptation. (**g**) Final crowns at the 3-year follow-up. Opened embrasure spaces for enhanced interproximal plaque control. (**h**) Radiographic evaluation at the last follow-up (3-year) presents stable crestal bone level in both test and control sites.

After appropriate anesthesia of the surgical area, full-thickness flap was elevated, including intrasulcular incisions of at least one neighboring tooth. Drilling was performed according to the manufacturer’s protocol using drills with stops at the relevant length (C-1 MIS Implants Technologies, Bar Lev industrial zone, Misgav, Israel). All implants were C-1, which combines an antirotational six-position cone index (conical abutment) and a platform switch design. The implant shoulder was flushed with the bone (Figure 1 and Figure 2a). Each patient received a pair of C1 implants placed next to each other. To minimize the influence of the operator on the surgical procedures between the groups, randomization of the implants to test or control was performed according to a predetermined computer-generated randomization scheme (Excel, using the index function, random between DEFs to HEAs). This was done only after final seating of the implants implemented with a torque-controlled ratchet. An insertion torque ≥35 N cm was considered to indicate sufficient primary stability.

**Figure 3:**
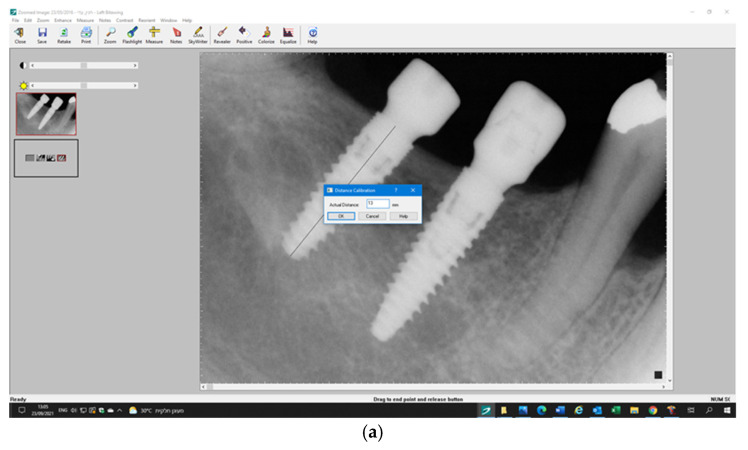

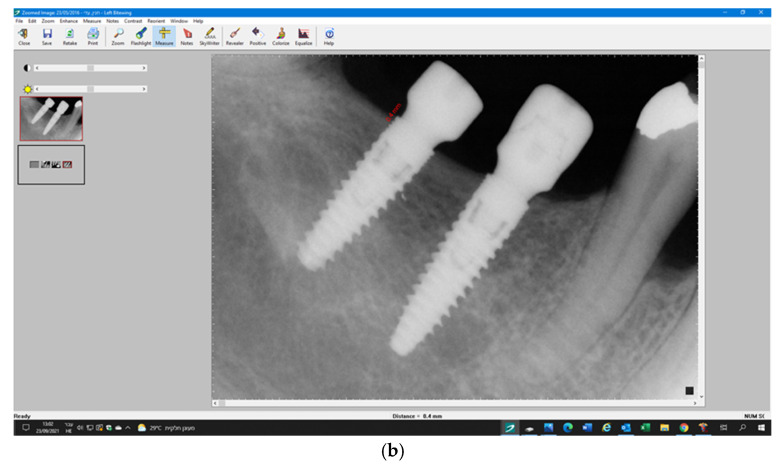
Radiographic measurements. (**a**). Radiographic distortion was calculated by dividing the radiographic implant length by the precise implant length using the measure (line) and calibration function. (**b**). Marginal bone level (MBL) was measured from the first bone-to-implant contact and implant shoulder.

One of the two implants was connected to a multiunit abutment (test) and the other was connected to a healing abutment (control) (Figure 2b). The multiunit abutment (1–3 mm gingival height) was tightened at 15 N cm and covered with a cover device and was not disconnected during the rehabilitation process. The other implant was connected to a 4–6 mm healing abutment. After implant placement and connection of the HEAs or DEFs an orthoradial periapical X-ray was performed (Figure 1 and Figure 3a). The flaps were adapted using simple interrupted absorbable 4/0 sutures (Vicryl rapid, Ethicon, Johnson and Johnson, Somerville, NJ, USA).

Postsurgical treatment included the same antibiotic administration (Augmentin 875 mg × 2 per day or Dalacin-C 150 mg × 4 per day for penicillin-allergic patients) for one-week postsurgery and 4 mg dexamethasone administered for 2 successive days. Pain relief analgesics included 275 mg naproxen sodium (Narocin, Teva Pharm Ind., Ltd., Petah Tikva, Israel) as needed. The patients were instructed to rinse with 0.2% chlorhexidine twice daily for 2 weeks, starting the day after treatment. Patients were instructed to refrain from mechanical plaque removal in the treated area of for 1 week. Suture removal and hygiene training were performed after 10–14 days.

### 3.2. Prosthetic Procedures

After a healing time of 3–6 months (Figure 2c,d) (as dependent if implants were placed in the maxilla or mandible), impressions were taken using putty and silicone washes (Express, 3M ESPE dental products, St. Paul, MN, USA) and interarch relations were recorded (Table 1). The impressions were performed using the open-tray technique (“Sandwich”) after adaptation of color-coded transfers (Figure 2e) that were verified radiographically (Figure 1b and Figure 2f) (first removal of the healing abutment). Due to the placement of the implants in nonaesthetic areas, temporary crowns were not performed. A master model with silicon imitation of marginal gingiva was prepared by the technician. At the following appointment, abutments were connected (second removal of the healing abutment), and the metal framework was tried. At this stage, a silicone pick-up impression of the metal framework in situ was taken. The permanent porcelain fused to metal-fixed partial crowns were screwed using a torque-controlled prosthetic ratchet in the next meeting after minor occlusal adjustment and polished if needed (Figure 2c). In cases in which major discrepancies existed regarding the radiographic and clinical adaptation of the metal framework, a new impression was taken, or separation of the metal framework was done and then reconnection using pattern resin (Duralay Reliance Dental Manufacturing, Alsip, IL, USA). At the delivery appointment, the multiunit and internal connection abutments were retightened at 35 N cm according to the manufacturer’s protocol. In cases of discrepancies related to occlusal height, crowns contour, embrasure spaces, or contact points, one or two extra appointments were scheduled in which the healing abutment was removed.

### 3.3. Postoperative Follow-Ups and Treatment

Patients’ condition was maintained by a trained oral hygienist every 3 to 6 months (Figure 2g). Each visit comprised a clinical examination, oral hygiene instructions, deplaquing, and SRP wherever needed. Periapical radiographs were performed at 3–6 months after implant placement, before impressions were taken, and immediately after fixed ceramic crown delivery, followed by annual follow-up examinations (Figure 1c and Figure 2h).

### 3.4. Outcome Measurements 

The treatment outcomes included the following parameters:

Implant success rate was determined according to the criteria of Albreketsson and Zarb 1986 [22]: no pain; bone loss at first year <1.5 mm; annual bone loss <0.2 mm thereafter; no peri-implant radiolucency; no implant mobility; no signs of infection.

Implant-related complications:

- Peri-implant mucositis was defined as clinical signs of inflammation (BOP) with no bone loss [23].
- Peri-implantitis was defined as implants showing clinical signs of inflammation (BOP), increased PD (≥6 mm), and progressive bone loss of ≥3 mm [24].

Prosthetic characteristics and complications:

- Implant crowns bucco–palatal/lingual and mesiodistal diameter in mm measured using a caliper and a 1 mm periodontal probe.
- Multiunit gingival and transmucosal individual abutment height (in mm).

Mechanical complications related to loosening of abutments, loss of composite filling of the screw hall, or fracture of the porcelain bridge. Functional complications included cheek and lip biting.

Periodontal diagnosis and parameters at last recall visit:

▪ Plaque index (PI)—percentage of visible plaque measured at four sites per implant and tooth (mesial, midfacial, distal, and palatal) at the soft tissue margin [21]. The plaque was stained with a disclosing solution.
▪ Bleeding index—consisting of a dichotomous recording of the absence or presence of bleeding after probing the implant sulcus/pocket within 10 s after probing per site (mesial, midfacial, distal, and palatal).
▪ PD—measured using a light probing force (approximately 25 g) to the nearest mm using a periodontal probe (UNC 15, Hu-Friedy, Chicago, IL, USA). The probing depth was calculated per implant.
▪ Keratinized mucosal width (KMW) was measured using a 1 mm probe to the nearest mm (Hu-Friedy, Chicago, IL, USA).
▪ Gingival biotype (thin or thick) reflected by the transparency of the periodontal probe through the gingival margin.

### 3.5. Radiographic Measurements

Standardized radiographs, with parallel film (Kodak Ektaspeed plus, Eastman Kodak Co., Rochester, NY, USA) and the X-ray beam perpendicular to the implant were taken using plastic film holders (Dentsply-Rinn Corporation, Elgin, IL, USA). Marginal bone level (MBL) associated with the implants was evaluated using computerized digital radiography (Schick Technologies, New York, NY, USA) by two independent examiners (H.N. and R.K.). Evaluation was made by measuring the distance between the alveolar bone crest and the implant shoulder which served as a reference line (RL). The distance from the RL to the first bone-to-implant contact was measured on the mesial and distal sides of each implant. Radiographic distortion was calculated by dividing the radiographic implant length by the precise implant length using the measure (line) and calibration function (Schick Technologies, New York, NY, USA) (Figure 3a,b). The differences (ΔH) between the different time points and baseline measurements were calculated accordingly.

## 4. Statistical Analysis

Sample size was calculated to reveal a difference of 0.5 mm of marginal bone loss between the test and control sites with standard deviation of 0.76, and correlation of 0.5 (effect size 0.658) with 80% power and a 5% significance level. It was based on noncentral t-distribution. The statistical analysis was performed using SPSS version 25.0 (IBM, Armonk, NY, USA). The primary outcome variables were the marginal bone level (MBL) of the DEFs (test) vs. the HEAs (control) implants from the time of implant placement through prosthetic delivery time and to the 1-year and 3-year follow-up. ANOVA with repeated measures considering within- and between-subject factors compared the DEFs vs. the HEAs. The factors that were analyzed as correlated to MBL were time, PI, jaw (mandible/maxilla), periodontal diagnosis, bone quality, distance between implants (≥4 mm vs. ≤4 mm), and distance to neighboring teeth (≥1.5 mm vs. <1.5 mm). Other correlations were between implant length, diameter, width of keratinized gingiva (≥2 vs. <2 mm), height of abutment, mesiodistal width of implant crown, bucco/ lingual or palatal width of crown, and MBL. All tests were two-tailed, and a *p* value of ≤5% was considered statistically significant.

## 5. Results

A total of 21 consecutive patients, 6 men and 15 women, met the inclusion criteria (Table 2). The mean age was 66.23 ± 9.35 years at the time of implant placement (range 35–86). Three patients with well-controlled diabetes were included (HbA1c < 6.5). None of the patients was a smoker. Patient and implant characteristics are shown in Table 2. A total of 42 C-1 implants placed in healed sites included 21 connected to multiunit abutments (test) and 21 connected to healing abutment (control) that were disconnected 3–5 times in the control group. The distribution of implants according to diameter and length is listed in Table 3. The mean follow-up time was 36.85 ± 1.34 months (range: 34.5–40 months). The mean height of the transmucosal part of HEAs was 1.76 ± 0.78 vs. 2.19 ± 0.87 in the DEFs group.

### 5.1. Implant Survival and Success Rate

No implants were lost during the 3-year follow-up; thus, the survival and success rate was 100%. Peri-implant mucositis was noticed in 38.1% of DEFs and 41.9% of HEAs at the 3-year follow-up meeting.

### 5.2. Marginal Bone Level

**Figure 4:**
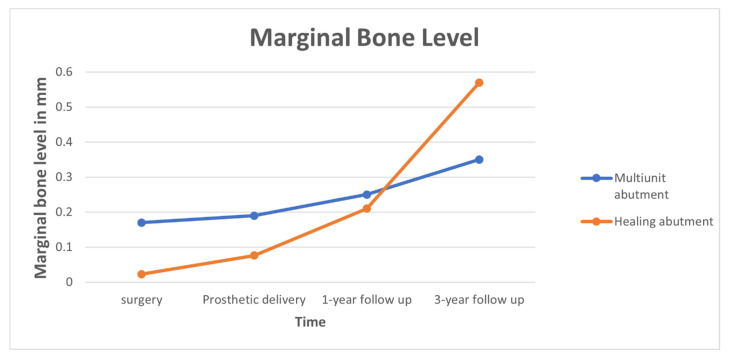
Marginal bone level in healing (red line) vs. multiunit (blue line) abutments at surgery and prosthetic delivery, and at the 1-year and 3-year follow-up evaluations.

Mean mesial and distal MBL at surgery, and prosthetic delivery at the 1-year and 3-year follow-up periods are presented in Figure 4 and Table 4. The comparison of mean marginal bone loss between the test and control groups was statistically significant at 3-year follow-up only (*p* = 0.005) (Table 3). A significant correlation existed between time and MBL in both groups (*p* = 0.000). The position of the implant and KTW did not affect MBL. No correlation was found between the periodontal parameters or prosthetic parameters described and MBL.

### 5.3. Mechanical Complications

Loosening of the abutment screw was diagnosed in one patient (4.7%). Retightening using a prosthetic ratchet was done. Loss of composite filling covering the screw hall occurred in two patients (9.5%). One female patient had cheek biting; this was solved by minimal selective grinding of the lower relevant teeth to increase the overjet dimension.

## 6. Discussion

### 6.1. Present Study Results

In the present study, we used SLA implants with conical connection and platform switch design, placed in healed sites with a delayed loading protocol. In general, the curves of bone level changes presented the same trend for both test and control groups. During the first period of up to 3–6 months, the changes in bone level were minimal in both groups as could be expected, since no disconnection had been performed in either group. No statistical correlation was found between periodontal parameters (plaque control and bleeding) or prosthetic characteristics and bone level, therefore the improved response in the DEFs implants at 3 years was not because of the better or worse control of plaque by the patient or due to prosthetic characteristics.

### 6.2. RCT—Similar Clinical Studies HEAs (Healing Abutments) vs. DEFs (Definitive Abutments)

The topic of our study was previously evaluated in different clinical scenarios including immediate postextraction implants [11,12,19] and healed sites [10,20,25,26,27]. The AOT protocol was further evaluated in immediately loaded implants with a definitive abutment [28]. Similar to the results in the present study, minimal differences existed between participants in the test group with definitive abutments (DEFs) and the control group with healing abutments (HEAs) in the short term at 4 months (−0.08 vs. −0.09 mm) [28] and 12 months (−0.06 vs. −0.23 mm) [29]. In the present study, the additive bone loss between the test and control group at the 3-year follow-up was 0.22 mm compared to 0.43 mm in the Bressan study [30]; although it was statistically significant in both studies, the clinical relevance of this difference may be questionable. Focusing on studies in which implants were placed in healed sites such as the present study [10,20,25,26,27,28], in three studies [20,26,27] statistically significant differences were not found in peri-implant bone loss between DEFs and HEAs. However, another study showed a significant additive bone loss of 0.63 (0.61 vs. 1.24 mm) 6 months after implant placement in favor of the DEFs groups [25].

### 6.3. Implant Neck Position Related to Crestal Bone

A significant factor to consider is the position of the implant neck platform related to crestal bone at the time of implant placement. Most studies in healed sites [20,25,26,27,28] described neck position at the crestal level (flush with bone level), although others [31,32] placed implants subcrestally considering that it would compensate for the expected remodeling of the ridge. However, in implants that were immediately placed [12,19,33], the position of the platform was subcrestal. In the present study, the implants were intended to be placed at the crestal level, although radiographic measurements revealed that the DEFs implants were placed 0.17 mm subcrestally while the HEAs implants were placed 0.02 mm subcrestally. The placement of the implants at a slightly different depth as done in the present study reflects the fact that alveolar ridges are rarely flat, which makes it difficult to position them at the same level around the entire perimeter. Moreover, in daily practice, at the patient level there is no full control over the implant position.

### 6.4. Platform Switch Design

One of the logical reasons that could explain the results of the current study and most of the cited data showing that no differences were found between the two protocols, is that most studies were performed using platform-switching designed restorations that from the outset may better preserve crestal bone levels. Thus, in platform-switch designed implants, the expected bone loss is minimal as previously confirmed by the results of a systematic review [17].

### 6.5. Abutment Type

In addition to the previously mentioned design of the abutments, the type of connection between the implant and the abutment determines relevant differences in peri-implant marginal bone loss. Implants with internal conical connections, such as implants used in the present study, had less bone loss compared to implants with external connections such as external hexagons [34,35]. Thus, it is possible that other variations inherent to each implant model exert influence on this outcome [36]; therefore, a careful analysis would be required to infer the results of this study and apply them to other implant models.

### 6.6. Time of Prosthetic Loading

Another cofactor that can affect bone remodeling and consequently the result of the study is the time of prosthetic loading. Both test and control implants received crowns at the same time (4-month mandible and 7-month maxilla) thus the only difference was that, during this period, in the HEAs group the healing screw was removed 3–5 times.

### 6.7. Abutment Height and Emergence Profile

The variation in divergence, width, and height of the prosthetic or healing abutments may result in a closer proximity between the abutment edge and the bone crest. This could stimulate bone resorption by compression of the ridge, or by the need to develop a biological width. In our study, as opposed to previous studies [37,38,39], no difference was found when comparing peri-implant bone loss in relation to the height of the multiunit (test) or transmucosal part of superstructures (control). Galindo [37] observed better results regarding peri-implant bone loss in implants that were placed with abutments of more than 2 mm in height compared to abutments of ≤2 mm after an 18-month follow-up period. Blanco [38] compared abutments of 1 vs. 3 mm and found differences both at 3 months (0.83 vs. 0.14 mm) and at 6 months of follow-up (0.91 mm vs. 0.11 mm). Novoa [39], comparing abutment heights of 1 vs. 2.5 mm, concluded that peri-implant bone loss was greater in those patients in which short abutments were used (1.30 vs. 0.33 mm) at 36-month follow-up. Another study confirmed that the shorter the abutment height, the greater the marginal bone loss in cement-retained prostheses [40]. In contrast to the previous studies described, a recent study failed to detect differences between 1- and 2-mm height abutments over a 1-year follow-up period [41]. In the present study, the multiunit abutment height was chosen to fulfill aesthetic demands in the maxilla when applied (1 mm sub gingivally) or at the gingival level in mandibular cases. Moreover, it was found that a wider and divergent emergence profile led to an apical displacement of the peri-implant biological space and greater bone loss [42]. Similarly, the heights of transmucosal part of superstructures were chosen following the same criteria at the time of impression taking. In the present study, the mean height of the transmucosal part of the superstructure in the HEAs group (control) was 1.76 ± 0.78 vs. 2.19 ± 0.87 mm in the DEFs group (test), which may have contributed to the better preservation of the MBL in the test group.

### 6.8. Cementation vs. Screw Retained

In the present study we used screw-retained restorations. As already found, the presence of excess cement may have a negative effect increasing bone loss [43].

### 6.9. Smoking

Another relevant aspect that could interfere with the results was the inclusion of smoking volunteers in all previous studies [11,12,19,20,25,29,30,33]. To avoid this type of influence [36], only nonsmokers were included in the present study.

### 6.10. Splinted Implants and Internal vs. External Connection

In the present study, implant crowns were connected to each other. Studies have suggested that splinted restorations offer load sharing among the components of the rehabilitation and decrease the stress on cortical bone [44]. At the same time, single-unit restorations (nonsplinted) facilitate oral hygiene, provide better passivity of the framework, and allow restorations with improved emergence profiles and cervical contours [44]. A recently published systematic review concluded that adjacent implants restored with splinted and nonsplinted fixed restorations did not differ in terms of crestal bone loss [45]. However, a statistical difference was found for implant survival rate, showing an advantage for splinted restorations [44]. Although internal and conical connection implants present lower values of bone loss than external connection implants, implants with platform switching, as used in the present study, showed lower bone loss values regardless of the connection type used, compared with platform-matched groups [34].

### 6.11. Clinical Relevance of the Study

The aim of the present study was to clinically verify the capability of the one-abutment at one-time protocol to prevent crestal bone loss in platform switch conical abutment implants that from the outset may better preserve crestal bone levels. The implants were followed for 36 months; thus, the data may add/fill the gap of knowledge in this field as only a few studies evaluated this protocol for 3 years.

### 6.12. Limitation of this Study

For aesthetic reasons, the selection of the height of the abutment is not random and is left to the discretion of who selects it, and this may condition possible crestal bone loss. Another factor that may impact the results is the variance in the emergence profile of the healing abutments or the definitive abutment in cases of premolars or molars. As previously mentioned, a wider and divergent emergence profile may lead greater bone loss [42]. However, due to the design of the implants next to each other, mainly in the posterior areas, this drawback could not be controlled.

### 6.13. Strengths of the Study

In this study, possible confounders were controlled by ensuring that all patients were nonsmokers and ensuring that implants were installed in healed sites. Porcelain restorations were manufactured by the same laboratory. Moreover, implants were placed in an optimal bacterial environment in very motivated patients. An additional strength is the design of the study, as the implants neighbored each other and were thus subjected to the same hygiene routines and maintenance program that equally affected both the test and control implants.

## 7. Conclusions

Statistically significant difference in marginal bone level was observed at the 3-year follow-up between the DEFs and HEAs groups. However, further clinical research is needed to clearly elucidate the real impact of abutment disconnection and reconnection, mainly in aesthetic implant-supported restorations. Since a time-dependent effect has been shown regarding bone remodeling, more randomized clinical studies with long-term follow-up are warranted. 

## Figures and Tables

**Table 1:** Schematic outline of the study.

Timeline	Preliminary Visit	Baseline Visit	12 Weeks	14–16 Weeks	24 Weeks	26–28 Weeks	1 Year	3 Years
Screen	x							
Admission criteria	x							
Informed consent	x							
Demographics	x							
Medical history	x							
Periodontal examination	x						x	x
Parallel periapical X-ray	x	x	x	x	x	x	x	x
SRP–hygiene reinforcement	x		x	x	x	x	x	x
Implant placement		x						
Impressions–maxilla					x			
Impressions–mandible			x					
Prosthetic delivery				x		x		

**Table 2:** Included patients and implants.

		No	%
Gender			
	Female	15	71
	Male	6	29
Implant position			
	Maxilla	9	43
	Mandible	12	57
Diabetes			
	No	18	86
	HbA1c < 7	3	14
Periodontal diagnosis			
	Stage 1	2	10
(Tonetti 2018)			
	Stage 2	6	29
	Stage 3	13	61
Bone Quality (Lekholm and Zarb 1985)			
	Type 1	2	10
	Type 2	12	57
	Type 3	7	33
Gingival biotype			
	Thick	17	81
	Thin	4	19
Implant site, maxilla			
	First premolar	4	9.5
	Second premolar	4	9.5
	First molar	7	16.5
	Second molar	3	7
Implant site, mandible			
	Central incisor	2	5
	First premolar	2	5
	Second premolar	3	7
	First molar	8	19
	Second molar	9	21.5

**Table 3:** Number of implants according to abutment and connection type (diameter and length in mm).

Abutment Type	Implant Diameter(mm)	8	10	11.5	13	16	Total
Multiunit							
	3.75	0	2	5	2	0	9
	4.2	1	2	3	2	1	9
	5	3	0	0	0	0	3
Healing cap							
	3.75	1	3	2	2	0	8
	4.2	1	1	2	3	0	7
	5	2	3	1	0	0	6
Total							42

**Table 4:** Differences (in mm) at the crestal bone level at surgery, prosthetic delivery, and 1-year and 3-year follow-up of the multiunit (test) and healing abutments (control).

	Multiunit Abutment (Test)		Healing Abutment (Control)	
	Mean ± SD, mm	Range, mm	Mean ± SD, mm	Range, mm
Surgery	0.17 ± 0.41	0–0.9	0.023 ± 0.076	0–0.3
Prosthetic delivery	0.19 ± 0.45	0–1.0	0.076 ± 0.17	0–0.5
1-year follow-up	0.52 ± 0.25	0–1.2	0.21 ± 0.41	0–1.0
3-year follow-up	0.35 ± 0.69	0–1.7	0.57 ± 0.80 *	0–1.7

* Statistically significant.

## Data Availability

Data supporting reported results can be found in the tables.
